# The highest oxidation state observed in graphene-supported sub-nanometer iron oxide clusters

**DOI:** 10.1038/s42004-023-00865-x

**Published:** 2023-04-03

**Authors:** Deborah Perco, Federico Loi, Luca Bignardi, Luca Sbuelz, Paolo Lacovig, Ezequiel Tosi, Silvano Lizzit, Aras Kartouzian, Ueli Heiz, Alessandro Baraldi

**Affiliations:** 1 Department of Physics, University of Trieste, Via Valerio 2, 34127 Trieste, Italy; 2 Elettra - Sincrotrone Trieste, AREA Science Park, 34149 Trieste, Italy; 3 Department of Chemistry, Technical University of Munich, Lichtenbergstrasse 4, 85748 Garching, Germany

**Keywords:** Nanoparticles, Characterization and analytical techniques, Surface spectroscopy

## Abstract

Size-selected iron oxide nanoclusters are outstanding candidates for technological-oriented applications due to their high efficiency-to-cost ratio. However, despite many theoretical studies, experimental works on their oxidation mechanism are still limited to gas-phase clusters. Herein we investigate the oxidation of graphene-supported size-selected Fe_n_ clusters by means of high-resolution X-ray Photoelectron Spectroscopy. We show a dependency of the core electron Fe 2p_3/2_ binding energy of metallic and oxidized clusters on the cluster size. Binding energies are also linked to chemical reactivity through the asymmetry parameter which is related to electron density of states at the Fermi energy. Upon oxidation, iron atoms in clusters reach the oxidation state Fe(II) and the absence of other oxidation states indicates a Fe-to-O ratio close to 1:1, in agreement with previous theoretical calculations and gas-phase experiments. Such knowledge can provide a basis for a better understanding of the behavior of iron oxide nanoclusters as supported catalysts.

## Abstract (web summary)

Iron oxide nanoclusters are of interest for a broad range of applications, but limited experimental information on their oxidation mechanism is available outside of the gas phase. Here, the oxidation of graphene-supported size-selected Fe_n_ clusters is studied using high-resolution X-ray Photoelectron Spectroscopy.

## Introduction

The quest for newly designed and efficient catalysts is a central issue that has attracted the interests of many researchers since it constitutes the essential stage to enhance the sustainability of many chemical processes of industrial relevance. In this respect, the possibility to prepare metallic-based nanostructures has provided a potentially groundbreaking way to improve the efficiency of traditionally designed catalysts and has been gaining interest in the scientific community in the last years^1,2^. Among the various nanostructured materials proposed and investigated, size-selected atomic clusters constitute a special class of objects whose properties depend strongly also on the number of atoms composing them and which have shown astounding catalytic performances^3–5^. The use of iron in these systems is of recent application. While Fe metal-based catalysts have been proven since long time to be crucial in industrially relevant reactions such as the Haber-Bosch process^6–9^, Fe atomic clusters stood out as potential candidate to substitute the highly expensive noble metal-based catalysts in the oxygen reduction reaction^10–13^, in the ammonia synthesis^14^ and for the alkene epoxidation^15^. The range of interest for Fe-based nanostructures further increases if we include Fe oxides^16,17^, which find several technological applications as magnetic storage media^18^, in biomedicine^19^ and as catalysts in several chemical reactions such as CO oxidation^20–24^, water splitting^25^ and in the Water Gas Shift^26^. Like their bulk counterparts, the properties of a specific Fe oxide cluster depend on its stoichiometry and oxidation state. For example, Fe_2_O_3_ cluster can oxidize CO to form CO_2_ and reduce NO to form N_2_ by undergoing compositional changes between Fe_2_O_2_ and Fe_2_O_3_ states^27^. A deep understanding of the intrinsic factors that determine the properties of oxide clusters is still lacking and it represents a hot topic in the scientific community as demonstrated by several theoretical results published in the last few years on Fe_n_O_m_ clusters^28–32^. While theory has highlighted the interest for these materials, experiments on the oxidation of Fe clusters are mainly limited to the gas phase^33–36^, which represents a fundamental approach for the understanding of these systems and for a comparison with theoretical works, but it is still far from the complexity of supported catalysts.

**Fig. 1:**
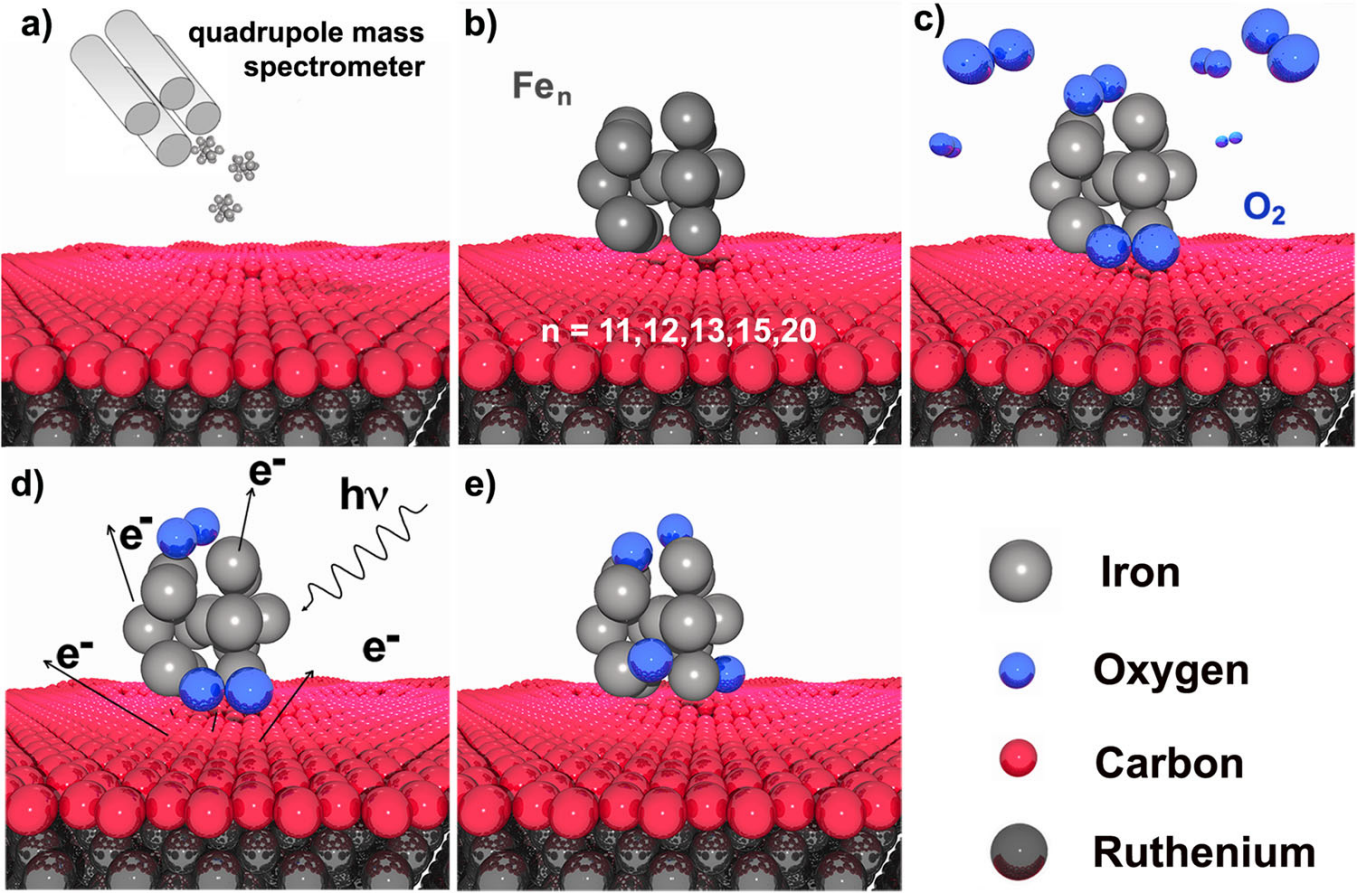
Outline of the oxidation process. **a** Fe clusters deposition on Gr/Ru(0001) **b** Adsorbed Fe cluster on the Gr/Ru(0001) interface. **c** O_2_ exposure at 20 K and physisorption on the Fe cluster at 20 K. **d** Emission of low-energy secondary electrons induced by soft x-ray irradiation. **e** Formation of atomic oxygen via dissociation of molecular oxygen and cluster oxidation.

In the present work, we studied the oxidation of size-selected Fe_n_ clusters with *n* = 11, 12, 13, 15 and 20 supported on graphene epitaxially grown on Ru(0001) by means of high-resolution X-Ray Photoelectron Spectroscopy with synchrotron radiation. Clusters were then oxidized with a photodissociation approach (Fig. 1). We have chosen to study Fe_13_ and Fe_15_ since they are magic clusters^37–39^ and have been already predicted to show enhanced stability and remarkable properties. At the same time, we intended to investigate some non-magic clusters whose size is larger and smaller than the two aforementioned magic ones, in order to showcase any remarkable differences. For example, recent density functional theory (DFT) calculations predicted that the 12 cluster shows an uncommon stability in the stoichiometry Fe_12_O_12_^40^. In this work we show that upon oxidation all the atoms in the supported Fe_n_ clusters can reach a maximum oxidation state equal to Fe(II), in agreement with theoretical works which predict a high stability for (FeO)_n_ clusters^41^. The differences between the stability of Fe(II) oxidation state in the nanocluster and in solid surfaces highlight the different behavior of iron oxides at sub nanoscale.

## Results and discussion

### Spectroscopic characterization of metallic Fe_n_ clusters

${{{{{{{{{\rm{Fe}}}}}}}}}_{{{{{{{{\rm{n}}}}}}}}}}^{+}$ nanoclusters with *n* = 11, 12, 13, 15 and 20 were deposited on graphene/Ru(0001), where they are electrically neutralized. Graphene/Ru(0001) is a very stable and highly corrugated 2D material which was already adopted for the growth and deposition of atomic clusters^42,43^. The morphology of this template reduces the cluster mobility as they tend to remain confined in the valley regions of the moiré lattice^44^. In fact, despite the calculated large adsorption energy of Fe adatoms on free standing graphene, *E*_a_ = 0.85 eV, the diffusion barrier is only 0.40 eV^45^, thus indicating that they are mobile at room temperature. To reduce the cluster mobility and to prevent sintering and nucleation, the temperature of the system was always kept at T = 20 K during the deposition and photoemission measurements. It is worth noting that the diffusion rate Γ on a surface follows the formula ${{\Gamma }}=\nu \ {\exp }^{-{{\Delta }}E/{k}_{{{{{{{{\rm{B}}}}}}}}}T}$, where *ν* is the vibrational frequency of the adatom, Δ*E* is the diffusion barrier, *k*_B_ is the Boltzmann constant and *T* the surface temperature. Using a prefactor *ν* equal to 10^13^, the diffusion rate that is obtained at room temperature is equal to 1.7 × 10^6^ s^−1^. On the other hand, at *T* = 20 K the rate becomes equal to 1.75 × 10^−88^ s^−1^, meaning that the diffusion of iron adatoms on graphene at a very low temperature is highly suppressed and become negligible on the time scale of our deposition and data acquisition.

**Fig. 2:**
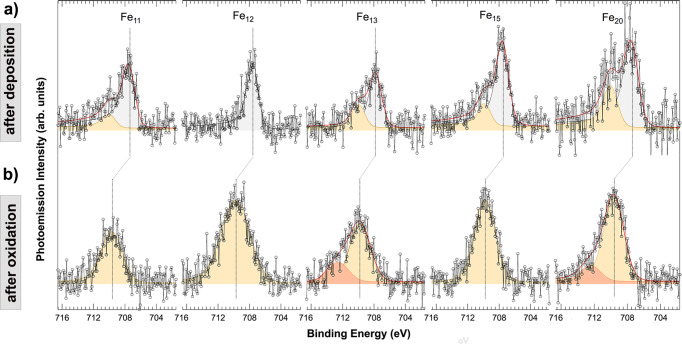
X-ray photoelectron spectra of as deposited and oxidized clusters. **a** Fe 2p_3/2_ core level spectra for as deposited and **b** oxidized Fe_11_, Fe_12_, Fe_13_, Fe_15_ and Fe_20_ nanoclusters supported on graphene/Ru(0001). Black markers and lines represent experimental data, the red line represents the best fit. Each peak stemming from the spectral analysis is shown in a different color. For the labeling of each peak please refer to the text.

The Fe 2p_3/2_ core level spectra of the supported pristine clusters are reported in Fig. 2a, together with the best fit and the spectral components. The very low cluster coverage on graphene (<0.1% ML), the low photoionization cross section and the large intrinsic broadening of 2p core levels (especially when compared to the 3d and 4f core levels, which are typically studied for heavier metals), make the data analysis particularly challenging. Such analysis, performed using Doniach-Šunjić functions^46^ convoluted with a Gaussian distribution, requires a rigorous procedure to obtain reliable information not only on the Fe 2p core level binding energies (BE) and of the number of non-equivalent components, but also of the core level photoemission line shape parameters, namely Gaussian (G) and Lorentzian (L) widths, and asymmetry (α). In this convolution, the Lorentzian lineshape L takes into account the core-hole lifetime through the uncertainty principles. The Gaussian width originates from several factors including the instrumental resolution, the phonon/vibrational broadening together with the inhomogeneous broadening due to the presence of a distribution of non-equivalent atomic configurations. The Anderson singularity index *α* describes the asymmetry of the lineshape due to the probability of electron-hole pairs excitation. The decision of using the Doniach-Šunjić function convoluted with a Gaussian distribution rather than the Voigt function, often preferred for quantitative analysis^47^, is justified by the fact that we are not interested in a composition analysis of the oxidized iron clusters, but in obtaining information about the asymmetry when it is present, as in clusters spectra before the oxidation process. Doniach-Šunjić functions convoluted with a Gaussian distribution have been extensively used in literature for fitting both metallic and oxidized surfaces^48,49^. Moreover, recently their use has been extended to properly fit the spectra of oxide nanoparticles and gas-phase nanoclusters^50,51^. Thus, we decided to fit XPS spectra of oxidized iron clusters using a Doniach-Šunjić function convoluted with a Gaussian distribution. This choice is motivated also by the results of Bano et al.^52^ which show that, on graphene, the HOMO-LUMO gap of iron oxide clusters is close to zero, allowing the use of this fitting function. This is due to a charge-transfer between graphene and clusters that involves also a semi-metal to metal transition of the graphene itself. Generally, it is important to point out that the Doniach-Šunjić function convoluted with a Gaussian distribution becomes a Voigt function when the value of *α* approaches to zero, as in our case. Since iron is a ferromagnetic material, the 2p_3/2_ core level measured on surfaces of single crystals is described using four components that mainly originates from exchange interactions^53–55^. However, since the signal-to-noise ratio in our spectra is low and since in literature the splitting between the components spans between 0.35 eV and 0.5 eV, we decided to fit our spectra using one single component. This choice is motivated also by the presence of a plethora of non-equivalent atoms in each cluster that would require using a number of components equal to 4n for the spectra of Fe_*n*_ clusters. The small error bar on the binding energy values of the different spectral components stemming from the fit, and reported in Table 1, is due to the complex fit procedure applied for interpreting the data and that is characterized by consecutive steps of analysis to extract the best fit parameters, as discussed in the Supplementary Note 1.

**Table 1:** BE in metallic and oxidized Fe-base materials.

Fe clusters			
Metallic		Oxide	
	BE (eV)		BE (eV)
Fe_11_	707.38 ± 0.10 ; 709.87 ± 0.10	Fe_11_	709.62 ± 0.10
Fe_12_	707.50 ± 0.10	Fe_12_	709.68 ± 0.10
Fe_13_	707.77 ± 0.10 ; 709.92 ± 0.10	Fe_13_	709.79 ± 0.10 ; 712.59 ± 0.10
Fe_15_	707.47 ± 0.10 ; 709.75 ± 0.10	Fe_15_	709.77 ± 0.10
Fe_20_	707.38 ± 0.10 ; 709.87 ± 0.10	Fe_20_	709.58 ± 0.10 ; 712.39 ± 0.10

Fe Bulk and Surfaces			
Metallic		Oxide	
	BE Fe^0^ (eV)		BE Fe^2+^ (eV)
Fe bulk	706.7^56^	FeO bulk	709.5^57^
Fe(100)/Cu(100)	706.5^57^	Oxidized Fe(100)/Cu(100)	709.2^57^
Fe(110)/Cu(100)	706.3^57^	Oxidized Fe(110)/Cu(100)	709.3^57^
Fe Polycrystal	706.28 ± 0.10	Oxidized Fe polycrystal	709.83 ± 0.10

Fe 2p_3/2_ core level BE for metallic and oxidized Fe clusters and polycrystal, as obtained from data analysis, and Fe bulk and surfaces, as reported in literature. Error analysis is described in Supplementary Note 1.

The spectral analysis in Fig. 2a indicates the presence of two different components for each of the investigated clusters besides Fe_12_. The position of the low BE component, which exhibits most of the spectral weight (Fig. 2a, gray curves), is affected non-monotonically by the cluster size, ranging from 707.38 eV for Fe_11_ and Fe_20_ up to 707.77 eV for Fe_13_ (Tab. 1). This component can be associated to Fe atoms in a metallic state. The shift towards higher BE with respect to Fe bulk (BE = 706.7 eV)^56^, single crystal surfaces (BE = 706.3 for Fe(110)/Cu(100) and 706.5 eV for Fe(100)/Cu(100)^57^) and to Fe polycrystal (BE = 706.28 eV, Supplementary Note 2) is in agreement with previous measurements on supported Pd and Au clusters on oxides^58,59^. For these systems, 3d and 4f core level shifts (CLS) were generally attributed to final state effects due to a size-dependent charging energy which scales with n^−1/3^ ^60^. However, the same model may not apply to our system, as the graphene/metal interface allows for charge transfer to the Fe clusters. A possible explanation to this experimental outcome may be linked to initial-state effects stemming from a lattice parameter contraction, which could significantly affect the CLS of the metallic Fe clusters, as proposed by Richter et al.^61^. Such guess appears to be reasonable since it is known that lattice strain causes a positive CLS: a strain of 6% in Cu, Ag and Au clusters composed of 13 atoms leads to a CLS of +0.79, +0.52 and +0.50 eV, respectively^62^. According to DFT calculations, the Fe − Fe distance in small Fe clusters in gas phase varies between 2.38 Å and 2.62 Å^37^ with a contraction in the range of 8 − 18% with respect to the value of iron bulk (2.86 Å^63^). The CLS that we report for iron nanoclusters range between +0.68 and +1.07 eV, thus in agreement with the general trend that enhanced contraction of the lattice parameter generates a larger CLS. An additional contribution to the shift could arise from the interaction of the cluster with graphene^64^ since the Fe-graphene bond strenght is not negligible, contrary, for example, to the case of Ag atoms^45^.

The agreement between the measured BE of the clusters and the expected CLS due to lattice strain suggests that the CLS in the clusters are dominated by initial state effects. Initial state effects are rich in chemical information as they depend also on the modifications of the d-band center, a well-known indicator of chemical reactivity^65^. For this reason, their dominant contribution to the overall CLS allows us to relate the reactivity of clusters with different sizes to their Fe 2p_3/2_ core level BE, with a low activity that can generally be associated to higher-than-expected BE, and vice versa^58^.

**Fig. 3:**
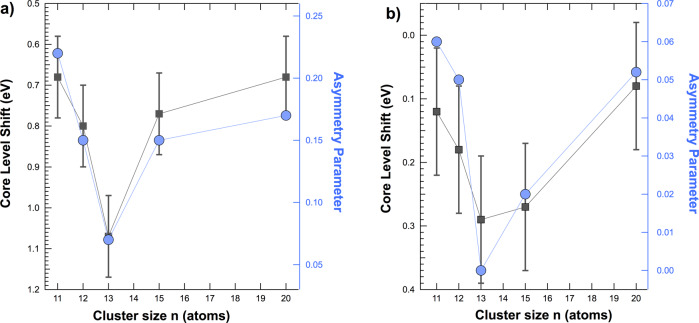
Comparison CLS vs asymmetry parameter. **a** Core level *α* parameters and CLS of metallic and **b** oxidized iron clusters as a function of their size. Circle markers refer to asymmetry parameter while square markers refer to CLS. The error bars on the core level shifts are of the order ± 0.1 eV.

In Fig. 3a (black markers) we report the CLS of the clusters Fe 2p_3/2_ core level with respect to the bulk value as a function of the cluster size. Fe_13_ shows the largest CLS (+1.07 eV), thus suggesting that this cluster is the most stable among the examined ones. Interestingly 13 is a magic number for Fe clusters associated to particularly stable configurations and, therefore, it is expected to show a low chemical reactivity^38,39^. The CLS trend we measured is in good agreement also with the trend of the asymmetry parameter *α* obtained from the spectral analysis. The *α* parameter in the Doniach-Šunjić function, is directly linked to the probability to excite single-electron excitations, namely electron-hole pairs, and it is hence related to the density of states near the Fermi level of the system^46^. It is interesting to note that a higher density of states near the Fermi level has been linked to an increased reactivity of the system increased^66^. The trend of *αvs*. the cluster size *n* is reported in Fig. 3a (blue markers) together with the CLS trend. The two curves match very well for all the cluster sizes, supporting our discussion on the relationship between clusters reactivity and CLS.

### Cluster oxidation and assignment of oxidation state

Nanoclusters oxidation was obtained employing a photodissociation approach at *T* = 20 K. At this temperature, it is possible to achieve a high degree of oxidation inducing the O_2_ dissociation by soft x-rays irradiation as schematically described in Fig. 1^43,67–69^. After being exposed to molecular oxygen (Fig. 1b), iron nanoclusters were irradiated with soft x-rays (Fig. 1c). We irradiated the sample for an overall exposure of 2 × 10^16^ photons s^−1^cm^−2^, with a X-ray photon energy of 805 eV for ca. 45 min. The photo-stimulation induces the production of secondary low-energy electrons thus resulting in O_2_ dissociation and atomic oxygen formation (Fig. 1d). The process of photoelectrons interaction with weakly bound physisorbed molecular oxygen results in intramolecular vibrational excitations via inelastic scattering. The effect has been proven using both photons and electrons, also by means of tunneling process employing scanning tunneling microscopy^70–72^. The oxidation of Fe nanoclusters results in a 2p_3/2_ CLS towards higher BEs (see Fig. 2b), confirming the well established trend found for Fe surfaces^73–75^. This component appears at the same BE of the low intensity peak revealed in the as-deposited clusters spectra (orange peaks in Fig. 2a), due to a partial oxidation of Fe cluster during deposition.

The Fe 2p_3/2_ core level spectra of oxidized Fe_11_, Fe_12_ and Fe_15_ nanoclusters can be fitted using a single component (Fig. 2b, yellow curve), while an additional component at higher BE (Fig. 2b, orange curve) was needed to obtain a low chi-square in the spectral analysis corresponding to the oxidized Fe_13_ and Fe_20_ clusters spectra. As happens for the BE of the metallic clusters, the Fe 2p_3/2_ BE of oxidized clusters is clearly affected by their size. The main component (yellow curve) ranges from 709.58 eV for Fe_11_ to 709.79 eV for Fe_13_ (Tab. 1). If compared to the metallic configurations, these BEs are closer to the Fe 2p_3/2_ core level of the bulk, surfaces and polycrystal Fe oxides, which are reported in Tab. 1. This is interpreted as a consequence of the Fe-O bond lengths in the clusters (1.81 − 1.96 Å)^41^ which become more similar to their bulk counterparts (1.98 Å for Fe_2_O_3_ and 2.09 Å for FeO^16^) than the Fe-Fe bond lenghts in the metallic case.

The asymmetry parameter *α* of the clusters decreases upon oxidation, going from the range 0.07–0.22 for metallic clusters down to 0.00 - 0.06. The trend of *α* still follows the CLS and it indicates that a higher chemical reactivity can be expected from oxidized clusters Fe_11_ and Fe_20_, which show the lowest CLS and the highest asymmetry (Fig. 3b). The same trend suggests that the least reactive oxidized clusters are Fe_13_ and Fe_15_, similarly to the metallic case. By comparison with previous XPS experiments, the main component in the oxidized spectra can be associate to Fe(II) ions (BE = 709.5 eV)^56,76^. Since our clusters are electrically neutral and all the Fe atoms possess the oxidation state 2 + , if we assume that O anions have the usual oxidation state 2 − , then we can conclude that the ratio between Fe and O atoms is close to 1:1. As a matter of fact, if we had a higher ratio, with more Fe than O atoms, we would expect to observe additional spectral components associated to Fe atoms with lower oxidation state or bonded with a lower number of O atoms. For example, the cage-like structure of the magic cluster Fe_13_O_8_ displays a Fe atom in its core in the metallic state^77^. Since from our spectra we can disregard the presence of atoms in a metallic state, we can exclude a similar Fe:O ratio. At the same time, if we had more O than Fe atoms in our clusters, we would expect to observe a component in the Fe 2p_3/2_ spectra compatible with Fe(III). The nominal BE value of the Fe(III) oxidation state is close to 711 eV^76^. The lack of compatible signal in the vicinity of this energy suggests that this oxidation state is most probably not present in the clusters. However, due to the different geometric and electronic properties of the clusters compared to bulk materials, the BE of the Fe(III) oxidation state could be shifted with respect to the nominal value^78,79^. Therefore, also considering the large FWHM of the spectra, it is not possible to unambiguously rule out the presence of Fe(III) species. However, these species would represent a minor fraction of the spectra, which are dominated by the Fe(II), supporting the conclusion that the ratio between Fe and O atoms is close to 1.

The formation of Fe(III) compounds has been ruled out because of the lack of compatible signal in the vicinity of BE = 711 eV^76^, which is the expected position of Fe(III) compounds in bulk materials. Although there might be differences in the position of the components in bulk and clusters, we observed that such differences are not so large for 2+ oxidation state (with a shift ranging from 0.08 up to 0.29 eV with respect to the value observed for bulk). Likewise, we do not expect larger differences for the peaks associated to the 3+ oxidation state. In addition to that, whereas there would be any Fe(III) components, they would constitute a negligible fraction, since their spectral weight is very small with respect to the overall signal.The interpretation of the component at higher BE observed for Fe_13_ and Fe_20_ oxidized clusters (BE = 712.59 eV and 712.39 eV, respectively) requires a further explanation. Although it has been proposed that a component at such binding energy could stem from the presence of FeCO_3_^56^, the formation of such compound would locally disrupt the graphene lattice on which the clusters are laying. On the other hand, it seems reasonable to assume that this component could originate from Fe atoms at the bottom of the cluster and interacting with the graphene layer underneath. Upon larger oxygen exposures, the Fe 2p_3/2_ spectrum does not further change, indicating that this configuration is associated to the highest oxidation state and oxygen coverage that the clusters can reach upon oxidation with atomic oxygen.

Our result for supported Fe clusters is also in agreement with theoretical predictions on the stability of the stoichiometry Fe_n_O_n_ for clusters of these sizes^41,80^, and with experimental findings on the oxidation of Fe clusters in the gas phase^33^. In particular, DFT calculations showed that Fe_12_O_12_ possesses unexpected stability and an extremely large band gap of 2.00 eV^40^. However, the same cluster shows several isomers separated only by few meV^80^. For example, the cage-shaped minimum energy configuration is favored by just 0.07 eV with respect of two tower structures composed of 3 × 3 and 4 × 4 rings which are nearly degenerate, with an energy difference of just 0.01 eV. The presence of different isomers can be read in our experiment from the Gaussian width (G value) obtained in the data analysis which is much larger than the overall experimental resolution. The oxidized Fe_12_ cluster shows the largest G value (4.12 eV) among the other oxidized clusters (G = 2.63 − 3.04 eV), thus suggesting that it could be the cluster with highest number of isomers, i.e., with a large distribution of non-equivalent local atomic configurations. We associate the increased G values of oxidized clusters with respect to the metallic ones (G = 1.51 − 1.83) to the larger number of non-equivalent local configurations that the oxygen adsorption leads to. As a matter of fact, given a certain number of metallic isomers which contribute to the Gaussian broadening for the spectra after deposition, each one can lead to several oxide structures for a specific oxygen density, thus raising the G width of the oxidized spectra. Finally, the larger G for the oxidized clusters can be affected by fluctuation of the 1:1 Fe to O ratio: clusters possessing a slightly different stoichiometry cannot be resolved in the spectral analysis and hence contribute to the overall spectral broadening.

Our investigation suggests that the oxidation process of supported iron nanoclusters is quite different from the case of iron solid surfaces. At room temperature, the oxide growth on iron surfaces typically involves several layers, it proceeds mainly via inward oxygen diffusion across the interface between metal-oxide and gas and it leads to the formation of two oxide phases. The first phase consists of a layer of FeO that has the function of a wet layer for the subsequent growth of a second oxide phase composed of Fe_2_O_3_^81^. Indeed, FeO on a surface tends to oxidize rapidly to form compounds with Fe ions in the Fe(III) oxidation state^73,82^. On the contrary, we show that the oxidation process of Fe nanoclusters leads to a stable Fe(II) oxide compound, as confirmed by negligible Fe 2p_3/2_ spectral changes upon further oxygen exposure. It is important to stress out that the oxidation method that we employed providing atomic oxygen at very low temperatures is extremely efficient, as it was proved for the case of Pt(111)^67^ and for the oxidation of size selected Ag clusters where atoms in the clusters reached an oxidation state 3+^43^. Conversely, the hindering towards a 3+ oxidation state in iron may be closely related to size of the unit cells of iron oxide compounds that include Fe(III) ions and higher density of oxygen atoms. For example, the unit cell of magnetite and maghemite accommodates 32 O^2−^ ions^16,83^, while there are only 4 in the unit cell of wüstite (FeO)^16^. We expect formation of such a large unit cell to be strongly unfavorable just because lattice deformations are energetically very expensive.

## Conclusions

In the present study, we investigated the oxidation of Fe_n_ nanoclusters with *n* = 11, 12, 13, 15 and 20 by means of high-resolution XPS. Metallic Fe clusters supported by Gr/Ru(0001) interface present a CLS towards higher binding energies with respect to iron bulk and solid surfaces. We attribute this phenomenon to lattice strain that generates a contraction of the Fe-Fe average interatomic distance of clusters with respect to bulk Fe. According to previous investigations, CLS are related also to the chemical reactivity of the system. In this respect we found that Fe clusters with larger CLS and lower asymmetry parameter are the most stable ones among the examined clusters. The BE of Fe clusters after the oxidation are more similar to bulk and surface oxide with respect to their metallic counterparts. The complete disappearance of the metallic component in Fe oxide clusters spectra paralleled by growth of a single new component at higher BE is interpreted as the formation of Fe clusters with a Fe to O ratio close to 1:1, in agreement with previous experiments of oxide clusters in gas phase. All Fe atoms in the clusters are in the Fe(II) oxidation state. Further oxygen exposures do not lead to any modification in Fe 2p_3/2_ core level spectra, suggesting that this is the most energetically favored configuration that clusters can reach upon photo-induced oxidation at a very low temperature. The difference between the oxidation of iron bulk and surfaces and iron nanoclusters is a clear proof that matter at the sub-nanoscale behaves in a different way. We believe that our results can help shedding light into the oxidation process at the nanoscale and into the use of supported Fe oxide nanoclusters in chemical reactions.

## Methods

### Sample preparation

The Ru(0001) crystal was cleaned through several cycles of sputtering and annealing. Sputtering cycles were made using Ar^+^ ions (*E*_kin_ = 3 keV) while annealing cycles were made first in O_2_ atmosphere to remove C by raising the temperature each cycle, from 600 K up to 1100 K and finally by a flash annealing to 1570 K to induce the oxygen desorption. Graphene was grown by thermal decomposition of ethylene (C_2_H_4_) in two steps. In the first one, Ru crystal was heated to 1100 K and exposed to ethylene for 300 s at a pressure of 2 × 10^−8^ mbar. In the second step, Ru crystal was kept at 1100 K and exposed to ethylene for 900 s at a pressure of 5 × 10^−8^ mbar. The quality of the grown graphene was checked by looking at the Low Energy Electron Diffraction image (Supplementary Fig. 3a), which shows the extra diffraction spots of the (13x13) moiré pattern, and at the C 1s core level spectrum (Supplementary Fig. 3b), which displays the typical double components due to weakly and highly interacting regions of the unit cell^84^.

### Fe clusters depostion and oxidation

${{{{{{{{{\rm{Fe}}}}}}}}}_{11}}^{+}$, ${{{{{{{{{\rm{Fe}}}}}}}}}_{12}}^{+}$, ${{{{{{{{{\rm{Fe}}}}}}}}}_{13}}^{+}$, ${{{{{{{{{\rm{Fe}}}}}}}}}_{15}}^{+}$ and ${{{{{{{{{\rm{Fe}}}}}}}}}_{20}}^{+}$ clusters were generated by using ENAC (Exact Number of Atoms in each Clusters), the size-selected clusters source based on the laser ablation process and quadrupole mass spectrometer (Extrel 150QC RF-DC, mass range m/z 10–16000 amu) mass selection^85,86^ (Supplementary Fig. 4). Iron nanoclusters were deposited on graphene/Ru(0001) in a soft-landing regime, i.e. with a kinetic energy lower than 1 eV/atom to avoid clusters fragmentation^87^. The clusters coverage for each deposition was 0.06% ML, which ensures a statistical occupation of one iron cluster every 9 moiré cells, that is approximately one Fe cluster every 3000 carbon atoms. Depositions, chemical reactions, and measurements were performed at a temperature of 20 K to avoid clusters diffusion and nucleation.

### High-resolution X-Ray photoelectron spectroscopy

High- Resolution X-Ray Photoelectron Spectroscopy measurements were performed at the SuperESCA beamline of the synchrotron light source Elettra, Trieste (IT). Photoemission spectra of the core level region Fe 2p_3/2_ were recorded for each cluster, tuning the photon energy to generate photoelectrons with a kinetic energy of about 100 eV to enhance surface sensitivity. The overall energy resolution at the employed photon energy (*hν* = 805 eV) was 200 meV. The intensity of each photoemission spectrum was normalized to the photon flux, while the binding energy (BE) scale was accurately calibrated by measuring the Fermi energy of the substrate. Each component of the photoemission spectra was fitted using a Doniach-Šunjić^46^ function convoluted with a Gaussian distribution to account for experimental resolution, vibrational and inhomogeneous broadening. The analysis was performed using a polynomial background^88^ which empirically resulted to be the most appropriate for our spectra.

## Supplementary information

Supplementary Information

## Data Availability

The data presented in this study are available from the corresponding author upon reasonable request.
