# Supplementary material for: The highest oxidation state observed in graphene-supported sub-nanometer iron oxide clusters
Source: Commun Chem. 2023 Apr 3;6:61. doi: 10.1038/s42004-023-00865-x (PMC10070315; doi:10.1038/s42004-023-00865-x)
Supplement: Supplementary file 1 — Supplementary Information [file 42004_2023_865_MOESM1_ESM.pdf]

Supplementary Information

The highest oxidation state observed in graphene-supported sub-nanometer iron oxide clusters

Deborah Perco,[1] Federico Loi,[1] Luca Bignardi,[1] Luca Sbuelz,[1] Paolo Lacovig,[2] Ezequiel Tosi,[2] Silvano Lizzit,[2] Aras Kartouzian,[3] Ueli Heiz,[3] and Alessandro Baraldi[1,2,*]

[1]*Department of Physics, University of Trieste, via Valerio 2, 34127 Trieste, Italy.*

[2]*Elettra Sincrotrone Trieste, AREA Science Park, 34149 Trieste, Italy.*

[3]*Department of Chemistry, Technical University of Munich, Lichtenbergstrasse 4, 85748 Garching, Germany.*

[*] alessandro.baraldi@elettra.eu

SUPPLEMENTARY NOTE 1: DATA ANALYSIS DISCUSSION

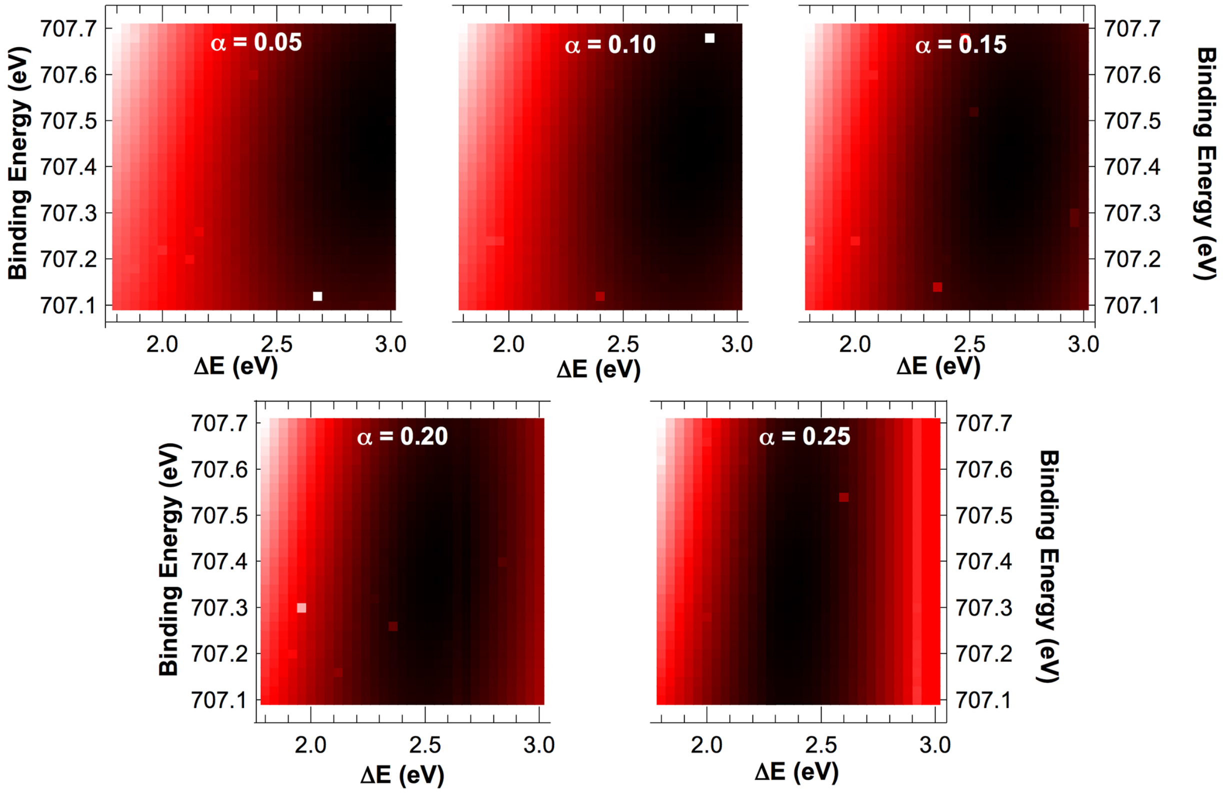

Supplementary Fig. 1. χ^2 **fitting analysis**. χ^2 trend as a function of BE and ΔE for different α parameters in th Fe $2p_{3/2}$ core level of metallic Fe_{15} cluster. Darker colours are associated to lower values of χ^2 while brighter colours are associated to higher.

To fit the Fe $2p_{3/2}$ spectra of metallic iron nanoclusters, components due to exchange splitting [1–3] were not considered. The splitting between the components of a photoemission peak due to exchange interactions is often of the same order of natural broadening, thus making difficult to separate different components in the data analysis. A custom-made data analysis method was used to analyze XPS spectra in a rigorous way. For each cluster, data analysis was divided in four steps and at the end of each step the value of one of the fitting parameters was determined. At the end of the first step, we determined the Gaussian (G) Full Width at Half Maximum (FWHM) parameter; after the second step, we determined the BE of the metallic or oxide components; at the end of the third step, we determined the asymmetry parameter α; at the end of the last step, we determined Lorentzian (L) FWHM parameter

and the shift (ΔE) between first and second component. In every step, the routine runs a double cycle (i.e. two cycles as defined in C language) in which the spectrum is fitted as a function of different values of two of the fitting parameters, while the other parameters are left free to vary. After every code run, χ^2 and fitting parameter values are saved. The best fit, for each run, is determined looking at the minimum value of χ^2. Eventually, for each step 2D color maps were created to evaluate the χ^2 trend varying fitting parameters. For example, in the first step, clusters spectra were fitted as a function of BE and ΔE values and the color maps obtained, varying α, are showed in Fig. 1 for Fe_{15} cluster. The error associated to a parameter value is evaluated computing the standard deviation of the values obtained for that parameter in each step and adding to this a systematic error that derives from the calibration of the photon beam and from the stability of the electronics of the energy analyzer.

SUPPLEMENTARY NOTE 2: OXIDATION OF POLYCRYSTALLINE IRON SLAB

To compare the results obtained for the oxidation of nanoclusters, we acquired the Fe 2p core level from a iron polycrystal (Mateck). After the cleaning process, consisting of Ar^+ sputtering for 45 minutes, the Fe 2p core level spectrum was acquired using a photon energy of 805 eV. Subsequently, the surface was oxidized using molecular oxygen at a low oxygen pressure, for a total exposure of about 50 Langmuir (L). After the oxidation, iron polycrystal Fe 2p spectrum was acquired again. Metallic iron polycrystal spectrum was fitted using three components, two for the $2p_{3/2}$ core level region (Fig. 2a, blue and green curves) and one for the $2p_{1/2}$ core level region (Fig. 2a, violet curve) while the oxidized iron polycrystal spectrum was fitted using four components, two for the $2p_{3/2}$ core level region (Fig. 2b, gray and green curves) and two for the $2p_{1/2}$ core level region (Fig 2b, violet and orange curves).

The BE of the polycrystal (BE = 706.28 eV) differs from the one of bulk iron (BE = 706.7 eV) but is very similar to the one of Fe(110) surface (BE = 706.3 eV). This is in agreement with the fact that bcc (110) surface of iron is the surface orientation that minimize the surface free energy [4, 5]. Moreover, the choice of a suitable photon energy, which is possible for experiments conducted at synchrotron radiation facilities, allows to have a high

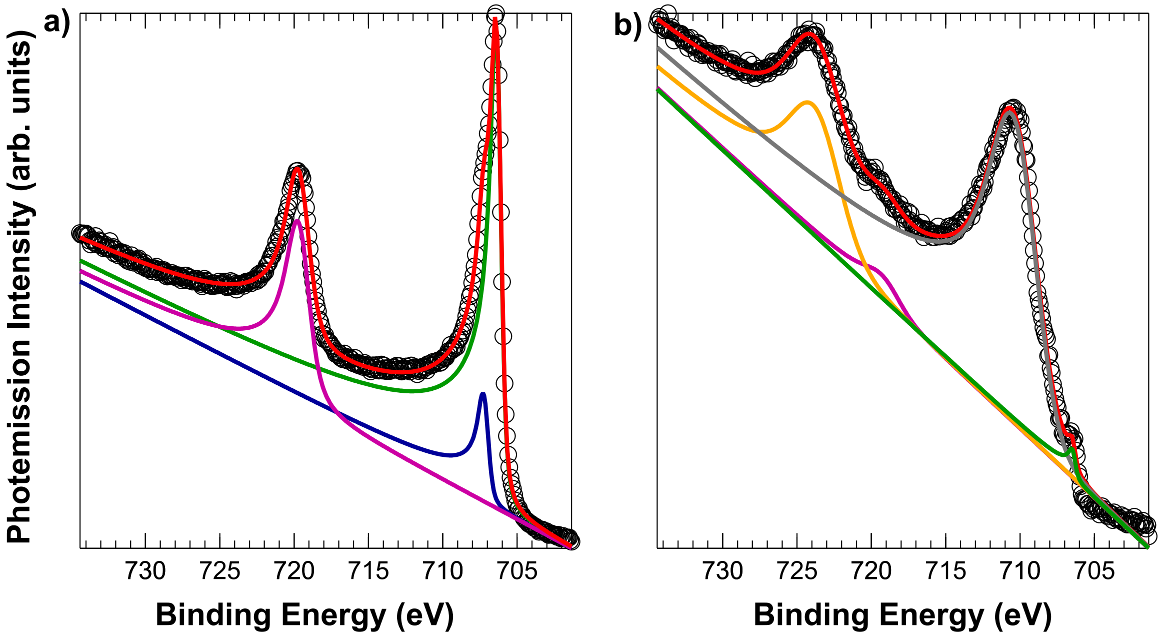

Supplementary Fig. 2. **Metallic and oxidized iron polycrystal.** a) Metallic and b) oxidized iron polycrystal Fe 2p spectrum together with the fit and the components obtained from the fit. Green and blue curves are associated to metallic Fe2$p_{3/2}$, violet curves are metallic Fe2$p_{1/2}$. In the oxidizes spectra, gray and orange curves are associated to oxidized Fe2$p_{3/2}$ and Fe2$p_{1/2}$, respectively

surface sensitivity. Therefore we observe that the contribution coming from Fe(110) surfaces dominate with respect to the one of bulk polycrystal and shifts the BE of 2$p_{3/2}$ core level. After the oxidation, the BE of the polycrystal is slightly higher (BE=709.83 eV) than the BE of bulk, surfaces and iron oxide clusters. This can be explained with the fact that on the surface a layer of Fe_3O_4 is forming. T. C. Lin et al. [6] indeed have found that, for oxygen exposures greater than or equal to 50 L, Fe(III) ions can be detected.

SUPPLEMENTARY FIGURES

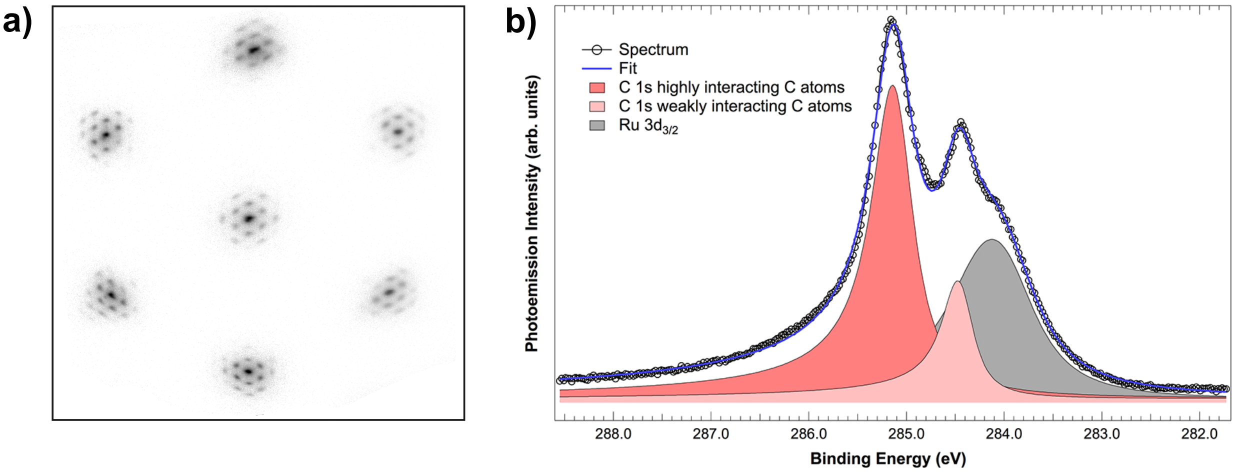

Supplementary Fig. 3. **Graphene characterization.** a) Spot profile analysis low energy electron diffraction pattern of the Gr/Ru surface aquired with electron energy E = 158 eV showing the moiré-induced diffraction spots. b) C 1s and Ru $3d_{3/2}$ core level spectrum. The red component at BE = 285.11 eV is associated with valley regions in the moiré pattern, while the pink component at BE = 284.43 eV is associated with hill regions. The gray component at BE = 284 eV is associated to Ru $3d_{3/2}$.

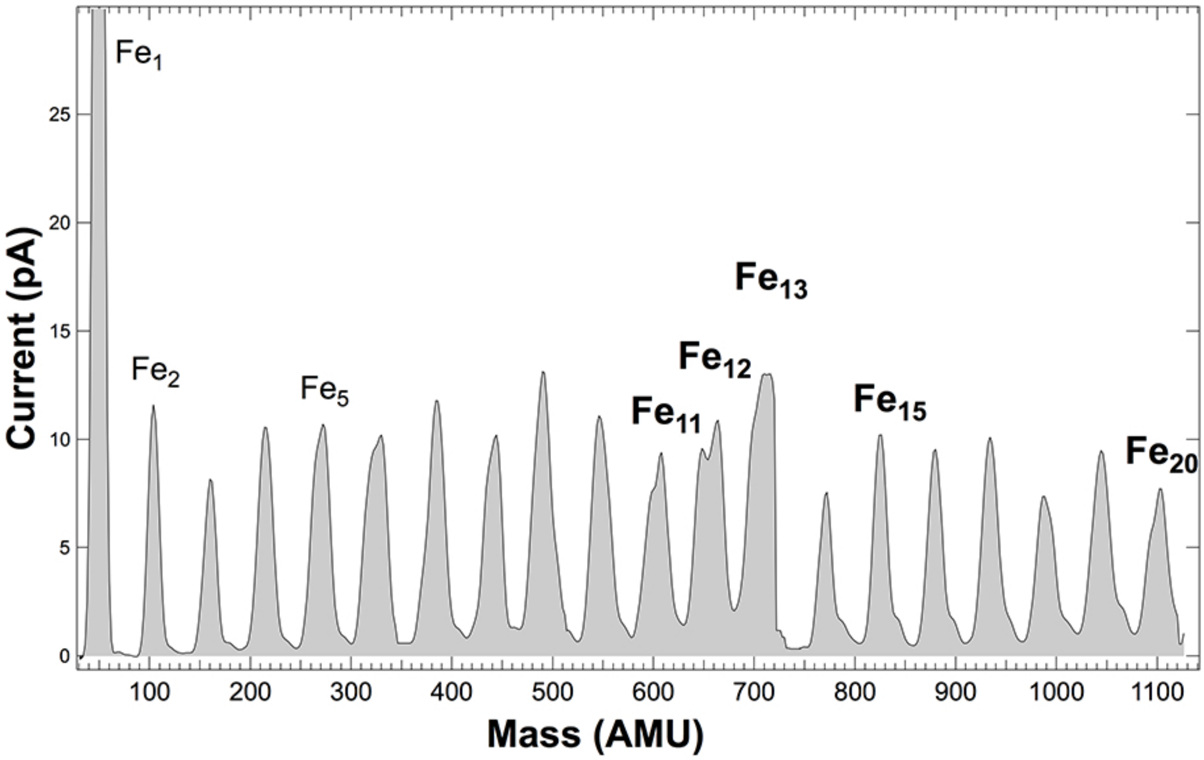

Supplementary Fig. 4. **Mass spectrum of size-selcted $\mathbf{Fe_n}^+$ clusters** Mass spectrum of Fe_n^+ clusters in the gas phase with n= $1-20$ obtained with the cluster sourc ENAC.

SUPPLEMENTARY REFERENCES

[1] O'Handley, R. Magnetic Materials in Encyclopedia of Physical Science and technology, Third Edition (ed. Meyers, R. A.), 919–944 (Academic Press, 2003).

[2] Cherepkov, N. A. Origin of magnetic dichroism in angular-resolved photoemission from ferromagnets. *Phys. Rev. B* **50**, 13813–13816 (1994).

[3] Weissenrieder, J. *et al.* Oxygen structures on Fe(110). *Surf. Sci.* **527**, 163–172 (2003).

[4] Schönecker, S., Li, X., Johansson, B., Kwon, S. K. & L, V. Thermal surface free energy and stress of iron. *Scientific Reports* **5**, 14860 (2015).

[5] Blonski, P. & Kiejna, A. Structural, electronic, and magnetic properties of bcc iron surfaces. *Surf. Sci.* **601**, 123–133 (2007).

[6] Lin, T. C., Seshadri, G. & Kelber, J. A. A consistent method for quantitative xps peak analysis of thin oxide films on clean polycrystalline iron surfaces. *Appl. Surf. Sci* **119**, 83–92 (1997).
